# Supplementary material for: Storage strategy of outbound containers with uncertain weight by data-driven hybrid genetic simulated annealing algorithm
Source: PLoS One. 2023 Apr 7;18(4):e0277890. doi: 10.1371/journal.pone.0277890 (PMC10081779; doi:10.1371/journal.pone.0277890)
Supplement: Attachment — Submitted filename: comment.docx [file pone.0277890.s001.docx]

PONE-D-22-19090

Storage Strategy of Outbound Containers with Uncertain Weight by Data-driven Hybrid Genetic Simulated Annealing Algorithm

PLOS ONE

Dear Dr. wang,

Thank you for submitting your manuscript to PLOS ONE. After careful consideration, we feel that it has merit but does not fully meet PLOS ONE’s publication criteria as it currently stands. Therefore, we invite you to submit a revised version of the manuscript that addresses the points raised during the review process.

Please submit your revised manuscript by Sep 22 2022 11:59PM. If you will need more time than this to complete your revisions, please reply to this message or contact the journal office at [plosone@plos.org](mailto:plosone@plos.org). When you're ready to submit your revision, log on to <https://www.editorialmanager.com/pone/> and select the 'Submissions Needing Revision' folder to locate your manuscript file.

Please include the following items when submitting your revised manuscript:

- A rebuttal letter that responds to each point raised by the academic editor and reviewer(s). You should upload this letter as a separate file labeled 'Response to Reviewers'.
- A marked-up copy of your manuscript that highlights changes made to the original version. You should upload this as a separate file labeled 'Revised Manuscript with Track Changes'.
- An unmarked version of your revised paper without tracked changes. You should upload this as a separate file labeled 'Manuscript'.

If you would like to make changes to your financial disclosure, please include your updated statement in your cover letter. Guidelines for resubmitting your figure files are available below the reviewer comments at the end of this letter.

If applicable, we recommend that you deposit your laboratory protocols in protocols.io to enhance the reproducibility of your results. Protocols.io assigns your protocol its own identifier (DOI) so that it can be cited independently in the future. For instructions see: <https://journals.plos.org/plosone/s/submission-guidelines#loc-laboratory-protocols>. Additionally, PLOS ONE offers an option for publishing peer-reviewed Lab Protocol articles, which describe protocols hosted on protocols.io. Read more information on sharing protocols at <https://plos.org/protocols?utm_medium=editorial-email&utm_source=authorletters&utm_campaign=protocols>.

We look forward to receiving your revised manuscript.

Kind regards,

Seyedali Mirjalili
Academic Editor
PLOS ONE

Journal Requirements:

When submitting your revision, we need you to address these additional requirements.

1. Please ensure that your manuscript meets PLOS ONE's style requirements, including those for file naming. The PLOS ONE style templates can be found at 
<https://journals.plos.org/plosone/s/file?id=wjVg/PLOSOne_formatting_sample_main_body.pdf> and 
<https://journals.plos.org/plosone/s/file?id=ba62/PLOSOne_formatting_sample_title_authors_affiliations.pdf>

2. Please ensure that you include a title page within your main document. You should list all authors and all affiliations as per our author instructions and clearly indicate the corresponding author.

[Note: HTML markup is below. Please do not edit.]

Reviewers' comments:

Reviewer's Responses to Questions

**Comments to the Author**

1. Is the manuscript technically sound, and do the data support the conclusions?

The manuscript must describe a technically sound piece of scientific research with data that supports the conclusions. Experiments must have been conducted rigorously, with appropriate controls, replication, and sample sizes. The conclusions must be drawn appropriately based on the data presented.

Reviewer #1: Partly

Reviewer #2: Yes

Reply: Thank you for your suggestion. According to your suggestion, the data part of the current paper has been checked to confirm that the data in this paper is sufficient to support the relevant research content.

2. Has the statistical analysis been performed appropriately and rigorously?

Reviewer #1: No

Reviewer #2: No

Reply: Thank you for your reminder. According to your reminder, the content of the methods section and the results section has been revised, which improves the value of the research method in this paper.

3. Have the authors made all data underlying the findings in their manuscript fully available?

The [PLOS Data policy](http://www.plosone.org/static/policies.action#sharing) requires authors to make all data underlying the findings described in their manuscript fully available without restriction, with rare exception (please refer to the Data Availability Statement in the manuscript PDF file). The data should be provided as part of the manuscript or its supporting information, or deposited to a public repository. For example, in addition to summary statistics, the data points behind means, medians and variance measures should be available. If there are restrictions on publicly sharing data—e.g. participant privacy or use of data from a third party—those must be specified.

Reviewer #1: Yes

Reviewer #2: No

Reply: This is a good reminder. The research data and results analysis content of this article have been reviewed according to your reminder and confirmed that comprehensive data content has been submitted.

4. Is the manuscript presented in an intelligible fashion and written in standard English?

PLOS ONE does not copyedit accepted manuscripts, so the language in submitted articles must be clear, correct, and unambiguous. Any typographical or grammatical errors should be corrected at revision, so please note any specific errors here.

Reviewer #1: Yes

Reviewer #2: Yes

Reply: Thank you very much for your affirmation.

5. Review Comments to the Author

Please use the space provided to explain your answers to the questions above. You may also include additional comments for the author, including concerns about dual publication, research ethics, or publication ethics. (Please upload your review as an attachment if it exceeds 20,000 characters)

Reply: Thanks for your affirmation. The article has been optimized more comprehensively, and I hope the article can be published.

Reviewer #1: The topic of this manuscript is interesting, however, the following major issues should be addressed. I hope the authors will not be dispirited and these comments can help them to improve their manuscript.
Reply: Thanks for your careful review and suggestions. We have carefully revised the manuscript according to your comments.
1. The authors are recommended to revise the Abstract and consider the claim (s) and finding (s) of this study.

Reply: Thank you for your suggestion. Based on your suggestion, the content of the abstract has been revised to highlight the research and findings of this paper, thereby improving the plausibility of the abstract.
2. The novelty of this study is not clear, it is recommended to revise the Introduction by considering the novelty and importance of this study.

Reply: Thanks for your comment. The last paragraph of the introduction has been revised based on your comments, in which it highlights the novelty and importance of this study.
3. What is the superiority of your work compared to existing works? It is recommended to state the contributions of this study.

Reply: Thank you for your reminder. According to your reminder, the contributions of this research has been highlighted in the last paragraph of the introduction.
4. The property and constraints of the problem should be stated; please clarify that it is a single objective or multi-objective problem?

Reply: Thanks for your guidance. A discussion of the limitations of this study has been added at the end of the Conclusions section based on your guidance.
5. It is recommended to change the title of the section “Container stacking strategy based on Hybrid Genetic And SAA (HGASA)” to “Container stacking strategy based on hybrid genetic and simulated annealing algorithm (HGASA)”.

Reply: Thank you for your suggestion. Based on your suggestion, the chapter title "Container stacking strategy based on Hybrid Genetic And SAA (HGASA)" has been changed to "Container stacking strategy based on hybrid genetic and simulated annealing algorithm (HGASA)".
6. How do the authors are hybridized a discrete algorithm GA with continuous algorithm SA? Please clarify and added the stepwise of this hybridizing.

Reply: Thanks for your comment. The explanation of the mixing process of the algorithm in this paper has been highlighted in the content after Figure 6 based on your comments.
7. This study suffers from a fresh literature review. It is recommended to boost the literature review of this study by deep diving into recent optimizers like recent works, Starling murmuration optimizer: A novel bio-inspired algorithm for global and engineering optimization and QANA: Quantum-based avian navigation optimizer algorithm.

Reply: Thank you for your reminder. As per your reminder, Starling murmuration optimizer: A new bio-inspired algorithm for global and engineering optimization and QANA: Quantum-based avian Navigation optimizer algorithm have been cited and discussed.
8. There is a miss understanding of the data-driven concept and novelty of this study please clarify. The schematic of figure 1 is different from its description. Please rewrite the data-driven theory section.

Reply: Thanks for your guidance. The data-driven section has been rewritten in accordance with your guidance, and Figure 1 has been revised to make it more logical.
9. This study introduced a hybrid optimization algorithm and needs to address the recent hybrid and improved variants of metaheuristic algorithms like hybrid algorithms, Migration-based moth-flame optimization algorithm, and An improved moth-flame optimization algorithm with adaptation mechanism to solve numerical and mechanical engineering problems.

Reply: Thanks for your advice. Based on your suggestion, the design and use of the mothflame optimization algorithm has been added to the "Design of the hybrid genetic SAA" section.
10. The well- organization of this manuscript can increase its quality. Then it is recommended to consider the proposed method section ( 3. The proposed algorithm, 4. Experimental evaluation …).

Reply: Thanks for your comment. Sections such as “The proposed algorithm” have been modified based on your comments to make this article more plausible.
11. It is suggested to increase the understanding of Figure 3 by adding more details to it.

Reply: Thank you for your reminder. Based on your reminder, an understanding of the details of Figure 3 has been added, and the content in Figure 3 has also been optimized.
12. Change Lg in Eq. (1) to log. Also please check Eq. (3). The description of parameters is needed.

Reply: Thanks for your guidance. Lg in Equation 1 has been modified to log according to your guidance, and the factors in Equation 3 are also explained.
13. Eqs. 5, 6, and 7 are not clear.

Reply: This is a good reminder. Equations 5, 6, 7 have been modified and optimized according to your reminder.
14. The direction of some lines was not determined in Figures 6 and 10.

Reply: Thank you for your suggestion. The structures in Figure 6 and Figure 10 have been modified and optimized according to your suggestion.
15. The authors recommend providing a subsection of in-depth experimental analysis to investigate the impact analysis of claims. Also, the paper requires very deep analysis from different perspectives.

Reply: Thanks for your comment. Subsection headings have been added to the ‘Case analysis” section based on your comments to make this section more reasonable.
16. It is recommended to determine the overall effectiveness of the proposed algorithm using the effectiveness metric proposed and used in recent works, DMDE: Diversity-maintained multi-trial vector differential evolution algorithm for non-decomposition large-scale global optimization and Hybridizing of Whale and Moth-Flame Optimization Algorithms to Solve Diverse Scales of Optimal Power Flow Problem.

Reply: Thank you for your reminder. Based on your reminder, the moth flame optimization algorithm has been added at the end of the methods section to optimize the model. And the reference materials provided here are cited in the article.
17. It is recommended to consider the pseudocode of the proposed algorithm

Reply: Thanks for your guidance. Figure 11 provides part of the algorithm code, which has been added to the text according to your guidance.
18. Please consider a table and mentioned the description of all parameters.

Reply: Thank you for your suggestion. Table 1 has been added based on your suggestion where the specific parameters studied in this paper are provided.

Reviewer #2: The manuscript, in its present form, contains several weaknesses. Adequate revisions to the following points should be undertaken to justify the recommendation for publication.
1. The contribution is not stated also add at the end of the introduction section.

Reply: Thank you for your suggestion. The last paragraph of the Introduction has been revised based on your suggestion to add the contributions of the research in this paper.
2. All the sections and subsections must be included in the text, Such as :( Related theories and concepts, etc.).

Reply: Thanks for your guidance. Appropriate subsections have been added to the main text according to your guidance to make the content of this article more reasonable.
3. I recommend adding new meta-heuristic algorithms to the introduction section as a related work

Reply: Thanks for your opinion. Based on your comments, more algorithms have been added as technical references in the related work section.
4. The discussion of the results should be expanded. The conclusions should include what implications. this work has and what impact this research may have on the sector.

Reply: Thank you for your reminder. Based on your reminder, the discussion of the Results section has been expanded and discussed the strengths of the research in this paper, while also analyzing the implications of this paper and its implications for the industry in the conclusion.
5. I recommend redesigning the flowcharts with better quality

Reply: Thanks for your comment. All flowcharts have been optimized based on your comments to make it more reasonable.
6. the conclusion section is petite

Reply: This is a good suggestion. More content has been added to the conclusion section based on your suggestion and the conclusion section has been expanded.
7. The conclusion section has many weaknesses, it is strongly recommended to rewrite this section. also, please add future work to the conclusion section and discuss it briefly.

Reply: Thanks for your guidance. Research shortcomings and information about future research have been added in the Conclusions section based on your guidance.
8. Please add the system specifications used for the evaluation

Reply: Thank you for your reminder. Based on your reminder, Table 1 has been added and the specifications of the evaluation system are provided.
9. Please clarify that the problem space for optimizing this problem is discrete or continuous

Reply: Thanks for your careful reading. Section (5) has been added at the end of the Methods section based on your suggestion and provides the model optimization methods studied in this paper.
10. It is recommended that you share the code

Reply: Thank you for your suggestion. Part of the code for the model studied in this paper has been provided in Figure 11 based on your suggestion.

6. PLOS authors have the option to publish the peer review history of their article ([what does this mean?](https://journals.plos.org/plosone/s/editorial-and-peer-review-process#loc-peer-review-history)). If published, this will include your full peer review and any attached files.


If you choose “no”, your identity will remain anonymous but your review may still be made public.


**Do you want your identity to be public for this peer review?** For information about this choice, including consent withdrawal, please see our [Privacy Policy](https://www.plos.org/privacy-policy).

Reviewer #1: No

Reviewer #2: No

[NOTE: If reviewer comments were submitted as an attachment file, they will be attached to this email and accessible via the submission site. Please log into your account, locate the manuscript record, and check for the action link "View Attachments". If this link does not appear, there are no attachment files.]

While revising your submission, please upload your figure files to the Preflight Analysis and Conversion Engine (PACE) digital diagnostic tool, <https://pacev2.apexcovantage.com/>. PACE helps ensure that figures meet PLOS requirements. To use PACE, you must first register as a user. Registration is free. Then, login and navigate to the UPLOAD tab, where you will find detailed instructions on how to use the tool. If you encounter any issues or have any questions when using PACE, please email PLOS at [figures@plos.org](mailto:figures@plos.org). Please note that Supporting Information files do not need this step.

*In compliance with data protection regulations, you may request that we remove your personal registration details at any time.* [*(Remove my information/details)*](https://www.editorialmanager.com/pone/login.asp?a=r)*. Please contact the publication office if you have any questions.*

This message and any attachment are intended solely for the addressee and may contain confidential information. If you have received this message in error, please send it back to me, and immediately delete it. Please do not use, copy or disclose the information contained in this message or in any attachment. Any views or opinions expressed by the author of this email do not necessarily reflect the views of The University of Nottingham Ningbo China. This message has been checked for viruses but the contents of an attachment may still contain software viruses which could damage your computer system: you are advised to perform your own checks. Email communications with The University of Nottingham Ningbo China may be monitored as permitted by UK and Chinese legislation.
